# The chromosomal genome sequence of the spiny sea fan, *Muricea muricata* (Pallas, 1766) (Malacalcyonacea: Plexauridae) and its associated microbial metagenome sequences

**DOI:** 10.12688/wellcomeopenres.27232.1

**Published:** 2026-08-08

**Authors:** Carlos Prada, Jose Victor Lopez, Nicholas Jones, Nina Pruzinsky, Graeme Oatley, Elizabeth Sinclair, Eerik Aunin, Noah Gettle, Camilla Santos, Michael Paulini, Haoyu Niu, Victoria McKenna, Rebecca O’Brien, Wellcome Sanger Institute Tree of Life Management, Samples and Laboratory team, Wellcome Sanger Institute Scientific Operations: Sequencing Operations, Wellcome Sanger Institute Tree of Life Core Informatics team, EBI Aquatic Symbiosis Genomics Data Portal Team, Aquatic Symbiosis Genomics Project Leadership

**Affiliations:** 1 University of Rhode Island, Kingston, Rhode Island, USA; 2 National Coral Reef Institute and Department of Biological Sciences, Nova Southeastern University, Dania Beach, Florida, USA; 3 University Corporation for Atmospheric Research, Boulder, Colorado, USA; 4 NOAA Ocean Exploration, Silver Spring, Maryland, USA; 5 Tree of Life Programme, Wellcome Sanger Institute, Hinxton, England, UK

**Keywords:** Muricea muricata, spiny sea fan, genome sequence, chromosomal, Malacalcyonacea, microbial metagenome assembly

## Abstract

We present a genome assembly from a *Muricea muricata* specimen (spiny sea fan; Cnidaria; Anthozoa; Malacalcyonacea; Plexauridae). The genome sequence has a total length of 453.40 megabases. Most of the assembly (98.45%) is scaffolded into 16 chromosomal pseudomolecules. The mitochondrial genome has also been assembled, with a length of 19.29 kilobases. Gene annotation of this assembly by Ensembl identified 52 164 protein-coding genes. From the metagenome data, we recovered five bins, of which three were high-quality MAGs.

## Species taxonomy

Eukaryota; Opisthokonta; Metazoa; Eumetazoa; Cnidaria; Anthozoa; Octocorallia; Malacalcyonacea; Plexauridae; *Muricea*; *Muricea muricata* (Pallas, 1766) (NCBI:txid204328).

## Background

**Figure 1.**
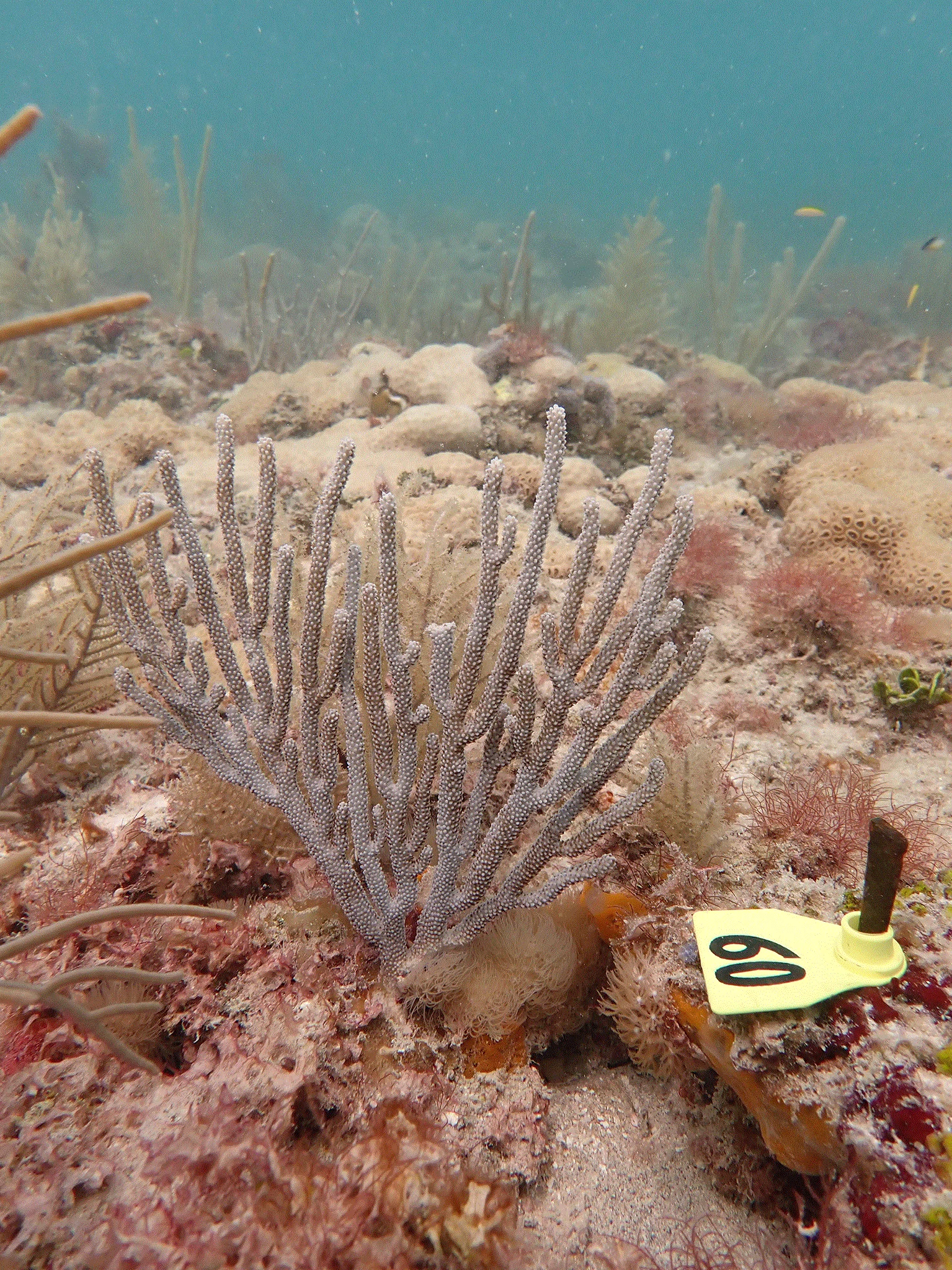
Photograph of a representative *Muricea muricata* specimen.

Gorgonians of the genus *Muricea* (Lamouroux, 1821) comprise about 30 nominal species worldwide, primarily distributed in the eastern Pacific with a few species occurring in the western Atlantic Ocean ( Bayer, 1961; Breedy & Guzman, 2016). *Muricea muricata* (Pallas, 1766) ( Figure 1) is one of the few representatives of the genus found in the Caribbean. It is commonly known as the “spiny sea fan”, due to its spiny, branching colonies with an arborescent growth form that extends into a fan-shaped colony. The branches are coated with a thick layer of living tissue called coenenchyme, which houses the coral’s polyps. Its colouration can range from light beige to dark orange or brown, depending on environmental factors and the presence of symbiotic organisms. The spines along its branches are a distinguishing feature, aiding in species identification and providing protection from certain predators ( Bayer, 1961).

*M. muricata* typically inhabits hard substrates in the photic zone, at depths of 3 to 30 m ( Collin *et al.*, 2005; SeaLifeBase, 2024). It prefers habitats with moderate to strong water currents, which facilitate nutrient and oxygen delivery essential for its survival. *M. muricata* establishes a symbiosis with dinoflagellates of the genus *Brevolium*, and it provides a food source for reef grazers and predators ( van Oppen *et al.*, 2005). Its branched structure also offers shelter and substrate for marine organisms, including small fish, crustaceans, and invertebrates ( Lasker *et al.*, 2020).

As a common member of Caribbean reefs, *Muricea muricata* contributes to the structural complexity of these systems, supporting diverse marine life while demonstrating remarkable resilience in the face of environmental changes. The density of *M. muricata*, like that of many other gorgonians, has increased despite the higher prevalence and intensity of marine heatwaves in tropical systems and the decline of many scleractinian coral populations ( Lasker *et al.*, 2020). *M. muricata* is, however, not immune to heat stress and, under unusually warm events, such as the 2005 event, is among the first gorgonians to show signs of bleaching ( Prada *et al.*, 2010).

As with other plexaurids, *M. muricata* has garnered attention in recent years due to its chemical ecology and adaptive mechanisms. Researchers have identified bioactive compounds within this species that may have pharmaceutical applications, including anti-inflammatory and antimicrobial properties ( Camacho *et al.*, 2014).

The nuclear genome assembly of *Muricea muricata* presented here could provide insights into the biology of this ecologically significant octocoral. Understanding its genetic blueprint would illuminate the molecular basis of its resilience to environmental stressors, such as heat stress and bleaching, offering clues to coral adaptation mechanisms amid climate change. This genome could reveal key genes involved in its unique growth strategies, branching patterns, and pseudo-vascular network, contributing to a broader knowledge of coral architecture.

Furthermore, the genome would serve as a vital resource for comparative studies, allowing researchers to explore the evolution of species after the formation of the Isthmus of Panama, as the genus *Muricea* is one of the few anthozoan genera with members on either side ( Breedy & Guzman, 2016). It could aid in identifying genetic factors underpinning the production of bioactive compounds, such as anti-inflammatory or antimicrobial metabolites, which have potential pharmaceutical applications.

By integrating the nuclear genome with ecological and physiological data, *M. muricata* could serve as a model species to better understand the genetic underpinnings of coral resilience and adaptation, ultimately contributing to coral reef preservation and restoration strategies.

## Methods

### Sample acquisition

*M. muricata* samples were collected at six sites in southeast Florida, USA between July and August 2021. At each site, at least three replicate colonies were tagged and sampled over four discrete time points (i.e., once before, twice during and once after the bleaching). Site depths ranged from ~10 to 20 m and were located offshore Miami-Dade (decimal degrees: 25.842, −80.104 and 25.842, −80.088), Central Broward (26.155, −80.089 and 26.159, −80.077) and Northern Broward (26.302, −80.068 and 26.301, −80.601). Each collection site was adjacent to fixed transects where the benthic community and temperature have been permanently monitored since 2003 and 2007 respectively as part of the Southeast Florida Coral Reef Evaluation and Monitoring Project (SECREMP). Samples were collected under Florida Fish and Wildlife Conservation Commission (FWC) recreational saltwater fishing licenses.

One of these samples was used for genome sequencing (specimen ID NSU0025901, ToLID jaMurMuri3). A second sample was used for Hi-C sequencing (specimen ID NSU0025902, ToLID jaMurMuri4). The jaMurMuri4 specimen was also used for RNA sequencing.

### Nucleic acid extraction

Protocols for high molecular weight (HMW) DNA extraction developed at the Wellcome Sanger Institute (WSI) Tree of Life Core Laboratory are available on protocols.io ( Howard *et al.*, 2025). The jaMurMuri3 sample was weighed and triaged to determine the appropriate extraction protocol. Tissue was homogenised by cryogenic disruption using the Covaris cryoPREP® Automated Dry Pulverizer. DNA was sheared into an average fragment size of 12–20 kb following the Megaruptor®3 for LI PacBio protocol. Sheared DNA was purified by manual SPRI (solid-phase reversible immobilisation). The concentration of the sheared and purified DNA was assessed using a Nanodrop spectrophotometer and Qubit Fluorometer using the Qubit dsDNA High Sensitivity Assay kit. Fragment size distribution was evaluated by running the sample on the FemtoPulse system. For this sample, the final post-shearing DNA had a Qubit concentration of 7.8 ng/μL and a yield of 3 042.00 ng.

RNA was extracted from tissue of the jaMurMuri4 specimen in the Tree of Life Laboratory at the WSI using the RNA Extraction: Automated MagMax™ *mir*Vana protocol. The RNA concentration was assessed using a Nanodrop spectrophotometer and a Qubit Fluorometer using the Qubit RNA Broad-Range Assay kit. Analysis of the integrity of the RNA was done using the Agilent RNA 6000 Pico Kit and Eukaryotic Total RNA assay.

### PacBio HiFi library preparation and sequencing

Library preparation and sequencing were performed at the WSI Scientific Operations core. Libraries were prepared using the SMRTbell Prep Kit 3.0 (Pacific Biosciences, California, USA), following the manufacturer’s instructions. The kit includes reagents for end repair/A-tailing, adapter ligation, post-ligation SMRTbell bead clean-up, and nuclease treatment. Size selection and clean-up were performed using diluted AMPure PB beads (Pacific Biosciences). DNA concentration was quantified using a Qubit Fluorometer v4.0 (ThermoFisher Scientific) and the Qubit 1X dsDNA HS assay kit. Final library fragment size was assessed with the Agilent Femto Pulse Automated Pulsed Field CE Instrument (Agilent Technologies) using the gDNA 55 kb BAC analysis kit.

The sample was sequenced on a Revio instrument (Pacific Biosciences, California, USA). Prepared libraries with a molarity greater than 2 nM were normalised to 2 nM, and 15 μL was used for making complexes. For libraries with a molarity below 2 nM, the available 10 μL library volume was used without normalisation. Primers were annealed and polymerases were bound to generate circularised complexes, following the manufacturer’s instructions. Complexes were purified using 1.2× SMRTbell beads, diluted to the Revio loading concentration of 200–300 pM, and spiked with a Revio sequencing internal control. The sample was sequenced on a Revio 25 M SMRT cell. SMRT Link software (Pacific Biosciences), a web-based workflow manager, was used to configure and monitor the run and to carry out primary and secondary data analysis.

### Hi-C

***Sample preparation and crosslinking***

The Hi-C sample was prepared from 20–50 mg of frozen tissue from the jaMurMuri4 sample using the Arima-HiC v2 kit (Arima Genomics). Following the manufacturer’s instructions, tissue was fixed and DNA crosslinked using TC buffer to a final formaldehyde concentration of 2%. The tissue was homogenised using the Diagnocine Power Masher-II. Crosslinked DNA was digested with a restriction enzyme master mix, biotinylated, and ligated. Clean-up was performed with SPRISelect beads before library preparation. DNA concentration was measured with the Qubit Fluorometer (Thermo Fisher Scientific) and Qubit HS Assay Kit. The biotinylation percentage was estimated using the Arima-HiC v2 QC beads.

***Hi-C library preparation and sequencing***

Biotinylated DNA constructs were fragmented using a Covaris E220 sonicator and size selected to 400–600 bp using SPRISelect beads. DNA was enriched with Arima-HiC v2 kit Enrichment beads. End repair, A-tailing, and adapter ligation were carried out with the NEBNext Ultra II DNA Library Prep Kit (New England Biolabs), following a modified protocol where library preparation occurs while DNA remains bound to the Enrichment beads. Library amplification was performed using KAPA HiFi HotStart mix and a custom Unique Dual Index (UDI) barcode set (Integrated DNA Technologies). Depending on sample concentration and biotinylation percentage determined at the crosslinking stage, libraries were amplified with 10–16 PCR cycles. Post-PCR clean-up was performed with SPRISelect beads. Libraries were quantified using the AccuClear Ultra High Sensitivity dsDNA Standards Assay Kit (Biotium) and a FLUOstar Omega plate reader (BMG Labtech).

Prior to sequencing, libraries were normalised to 10 ng/μL. Normalised libraries were quantified again to create equimolar and/or weighted 2.8 nM pools. Pool concentrations were checked using the Agilent 4200 TapeStation (Agilent) with High Sensitivity D500 reagents before sequencing. Sequencing was performed using paired-end 150 bp reads on the Illumina NovaSeq 6000.

### RNA library preparation and sequencing

Libraries were prepared using the NEBNext® Ultra II Directional RNA Library Prep Kit for Illumina (New England Biolabs), following the manufacturer’s instructions. Poly(A) mRNA was isolated from 100 ng of total RNA using oligo (dT) beads, converted to cDNA, and uniquely indexed; 14 PCR cycles were performed. Libraries were prepared with an intended fragment size of 100–300 bp. Libraries were quantified, normalised, and pooled equimolarly to a final concentration of 2.8 nM before dilution to 150 pM for loading. Sequencing was carried out on the Illumina NovaSeq X, generating paired-end reads.

### Genome assembly

Prior to assembly of the PacBio HiFi reads, a database of *k*-mer counts ( *k* = 31) was generated from the filtered reads using FastK. GenomeScope2 ( Ranallo-Benavidez *et al.*, 2020) was used to analyse the *k*-mer frequency distributions, providing estimates of genome size, heterozygosity, and repeat content.

The HiFi reads were assembled using Hifiasm ( Cheng *et al.*, 2021) with the --primary option. Haplotypic duplications were identified and removed using purge_dups ( Guan *et al.*, 2020). The Hi-C reads ( Rao *et al.*, 2014) were mapped to the primary contigs using bwa-mem2 ( Vasimuddin *et al.*, 2019), and the contigs were scaffolded in YaHS ( Zhou *et al.*, 2023) with the --break option for handling potential misassemblies. The scaffolded assemblies were evaluated using Gfastats ( Formenti *et al.*, 2022), BUSCO ( Manni *et al.*, 2021) and MERQURY.FK ( Rhie *et al.*, 2020).

The mitochondrial genome was assembled using MitoHiFi ( Uliano-Silva *et al.*, 2023). The MitoHiFi reference was the mitochondrial genome of *Muricea crassa* (NC_029697.1).

### Assembly curation

The assembly was decontaminated using the Assembly Screen for Cobionts and Contaminants ( ASCC) pipeline. TreeVal was used to generate the flat files and maps for use in curation. Manual curation was conducted primarily in PretextView and HiGlass ( Kerpedjiev *et al.*, 2018). Scaffolds were visually inspected and corrected as described by Howe *et al*. (2021). Manual corrections included 68 breaks, 69 joins, and removal of 48 haplotypic duplications. This reduced the scaffold count by 52.3%, increased the scaffold N50 by 303.9%, and reduced the total assembly length by 5.6%. The curation process is described at sanger-tol/curation-resources . PretextSnapshot was used to generate a Hi-C contact map of the final assembly.

### Assembly quality assessment

The Merqury.FK tool ( Rhie *et al.*, 2020) was run in a Singularity container ( Kurtzer *et al.*, 2017) to evaluate *k*-mer completeness and assembly quality for the primary and alternate haplotypes using the *k*-mer database ( *k* = 31) computed prior to genome assembly. The analysis outputs included assembly QV scores and completeness statistics.

The genome was analysed using the BlobToolKit pipeline, a Nextflow implementation of the earlier Snakemake version ( Challis *et al.*, 2020). PacBio reads were aligned to the assembly using minimap2 ( Li, 2018) and processed with SAMtools ( Danecek *et al.*, 2021) to generate coverage tracks. The pipeline runs BUSCO ( Manni *et al.*, 2021) using lineages identified from the NCBI Taxonomy ( Schoch *et al.*, 2020). For the three basal BUSCO lineages, eukaryota, bacteria and archaea, BUSCO genes are aligned to the UniProt Reference Proteomes database ( Bateman *et al.*, 2023) using DIAMOND BLASTp ( Buchfink *et al.*, 2021). The genome assembly is divided into chunks based on the density of BUSCO genes from the closest taxonomic lineage, and each chunk is aligned to the UniProt Reference Proteomes database using DIAMOND BLASTx. Sequences without DIAMOND BLASTx hits are extracted using seqtk and aligned to the NT database using BLASTn ( Altschul *et al.*, 1990). The outputs are consolidated into a BlobDir for visualisation in BlobToolKit. The BlobToolKit pipeline was developed using nf-core tooling ( Ewels *et al.*, 2020) and MultiQC ( Ewels *et al.*, 2016), with containerisation through Docker ( Merkel, 2014) and Singularity ( Kurtzer *et al.*, 2017).

### Metagenome assembly

The metagenome assembly was generated using MetaMDBG ( Benoit *et al.*, 2024). The resulting bin sets of each binning algorithm were optimised and refined using MAGScoT ( Rühlemann *et al.*, 2022). PROKKA ( Seemann, 2014) was used to identify tRNAs and rRNAs in each bin, CheckM ( Parks *et al.*, 2015) (checkM_DB release 2015-01-16) was used to assess bin completeness/contamination, and GTDB-Tk ( Chaumeil *et al.*, 2022) (GTDB release 214) was used to taxonomically classify bins. Taxonomic replicate bins were identified using dRep ( Olm *et al.*, 2017) with default settings (95% ANI threshold). All bins were assessed for quality and categorised as metagenome-assembled genomes (MAGs) if they met the following criteria: contamination ≤5%, presence of 5S, 16S, and 23S rRNA genes, at least 18 unique tRNAs, and either ≥90% completeness or ≥ 50% completeness with fully circularised chromosomes ( Bowers *et al.*, 2017). Bins that did not meet these thresholds, or were identified as taxonomic replicates of MAGs, were retained as ‘binned metagenomes’ provided they had ≥50% completeness and ≤ 10% contamination. A taxonomic tree of the bins was constructed from NCBI classifications using ete3 ( Huerta-Cepas *et al.*, 2016) and visualised with matplotlib.

## Genome sequence report

### Sequence data

**Figure 2.**
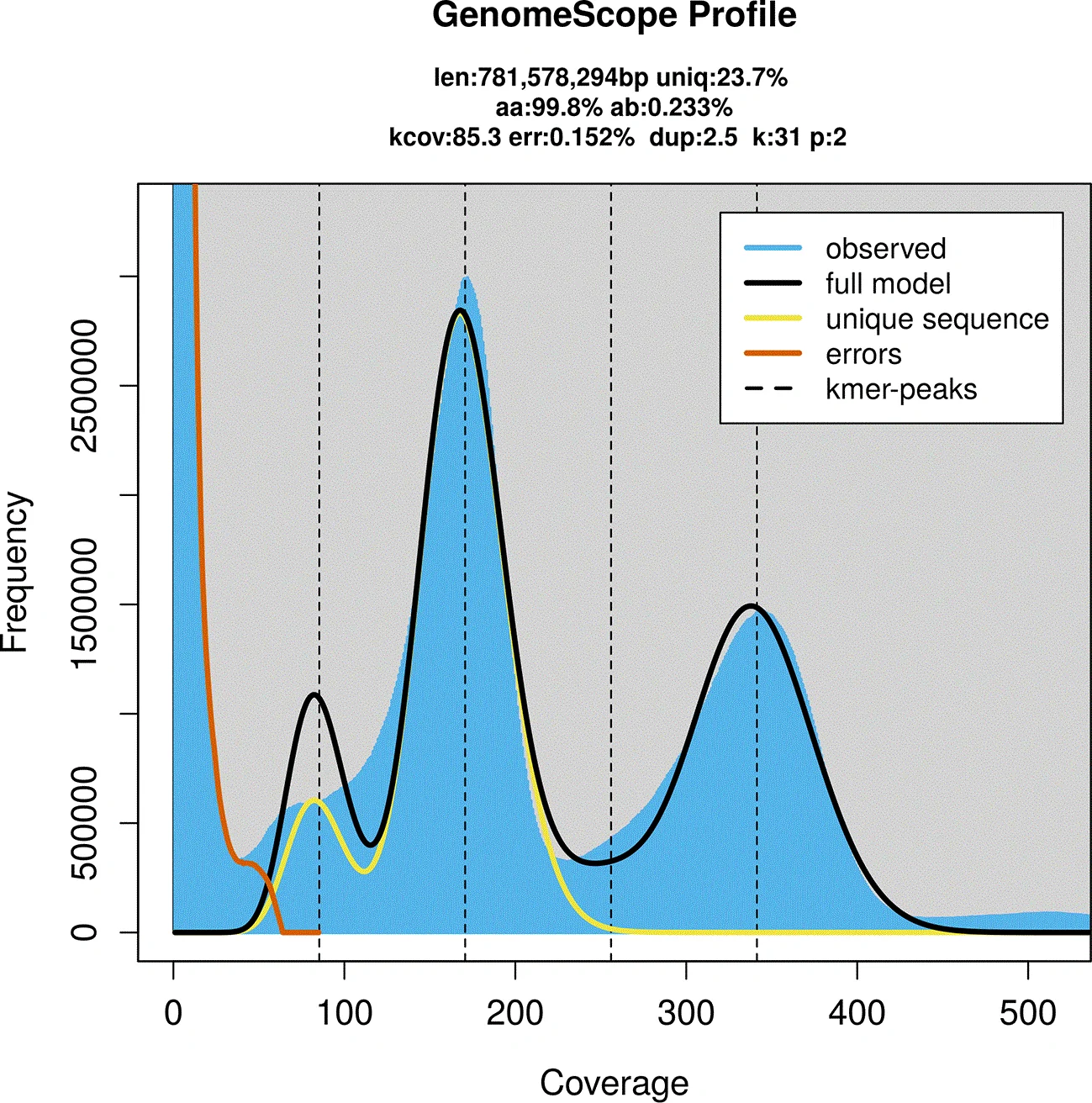
Frequency distribution of *k*-mers generated using GenomeScope2. The plot shows observed and modelled *k*-mer spectra, providing estimates of genome size, heterozygosity, and repeat content based on unassembled sequencing reads.

PacBio sequencing of the *Muricea muricata* specimen generated 144.63 Gb (gigabases) from 13.01 million reads, which were used to assemble the genome. GenomeScope2.0 analysis estimated the haploid genome size at 782.95 Mb, with a heterozygosity of 0.23% and repeat content of 76.28% ( Figure 2). These estimates guided expectations for the assembly. Based on the estimated genome size, the sequencing data provided approximately 118× coverage. Hi-C sequencing produced 83.84 Gb from 277.61 million reads, which were used to scaffold the assembly. RNA sequencing data were also generated and are available in public sequence repositories. Table 1 summarises the specimen and sequencing details.

**Table 1.** Specimen and sequencing data for *Muricea muricata* (BioProject PRJEB70484).

Platform	PacBio HiFi	Hi-C	RNA-seq
**ToLID**	jaMurMuri3	jaMurMuri4	jaMurMuri4
**Specimen ID**	NSU0025901	NSU0025902	NSU0025902
**BioSample (source individual)**	SAMEA12927197	SAMEA12927198	SAMEA12927198
**BioSample (tissue)**	SAMEA12927224	SAMEA12927227	SAMEA12927226
**Instrument**	Revio	Illumina NovaSeq 6000	Illumina NovaSeq X
**Run accessions**	ERR12319392; ERR12319393; ERR14749937; ERR15170261	ERR12321257	ERR13669958
**Read count total**	13.01 million reads	277.61 million read pairs	47.14 million read pairs
**Base count total**	144.63 Gb	83.84 Gb	14.24 Gb

### Assembly statistics

The primary haplotype was assembled, and contigs corresponding to an alternate haplotype were also deposited in INSDC databases. The final assembly has a total length of 453.40 Mb in 93 scaffolds, with 243 gaps, and a scaffold N50 of 60.66 Mb ( Table 2).

**Table 2.** Genome assembly data for *Muricea muricata.*

Genome assembly	Primary assembly
**Assembly name**	jaMurMuri3.1
**Assembly accession**	GCA_963855995.1
**Alternate haplotype accession**	GCA_963855965.1
**Assembly level**	chromosome
**Span (Mb)**	453.40
**Number of chromosomes**	16
**Number of contigs**	336
**Contig N50**	4.02 Mb
**Number of scaffolds**	93
**Scaffold N50**	60.66 Mb
**Organelles**	Mitochondrial genome: 19.29 kb

**Figure 3.**
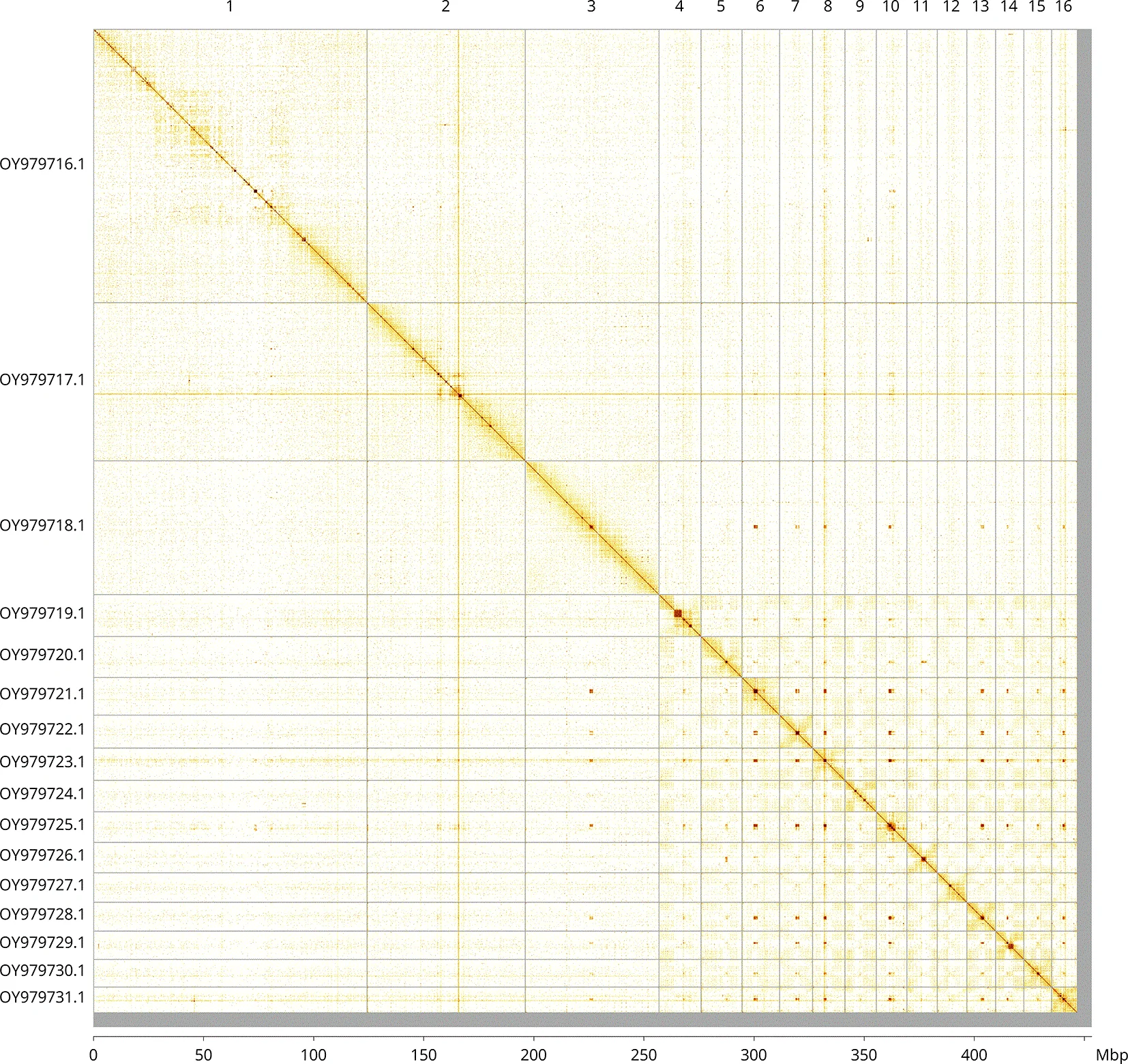
Hi-C contact map of the *Muricea muricata* genome assembly. Assembled chromosomes are shown in order of size and labelled along the axes, with a megabase scale shown below. The plot was generated using PretextSnapshot.

Most of the assembly sequence (98.45%) was assigned to 16 chromosomal-level scaffolds. These chromosome-level scaffolds, confirmed by Hi-C data, are named according to size ( Figure 3; Table 3).

**Table 3.** Chromosomal pseudomolecules in the primary genome assembly of *Muricea muricata* jaMurMuri3.

INSDC accession	Molecule	Length (Mb)	GC%
OY979716.1	1	124.19	36.50
OY979717.1	2	71.69	37
OY979718.1	3	60.66	36.50
OY979719.1	4	19.16	37
OY979720.1	5	18.58	36.50
OY979721.1	6	16.98	37
OY979722.1	7	15.02	37
OY979723.1	8	14.64	37
OY979724.1	9	14.29	37
OY979725.1	10	13.90	37
OY979726.1	11	13.74	37
OY979727.1	12	13.47	37
OY979728.1	13	13	37
OY979729.1	14	12.88	37
OY979730.1	15	12.57	37
OY979731.1	16	11.60	37.50

The mitochondrial genome was also assembled (length 19.29 kb, OY979732.1). This sequence is included as a contig in the multifasta file of the genome submission and as a standalone record.

### Assembly quality metrics

**Figure 4.**
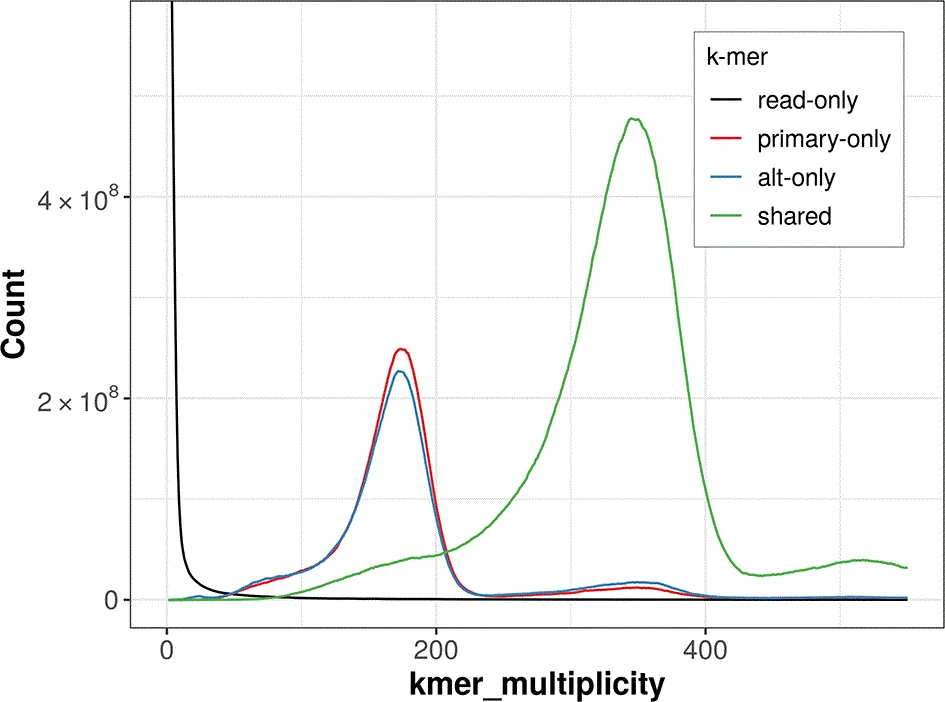
Evaluation of *k*-mer completeness using MerquryFK. This plot illustrates the recovery of *k*-mers from the original read data in the final assemblies. The horizontal axis represents *k*-mer multiplicity, and the vertical axis shows the number of *k*-mers. The black curve represents *k*-mers that appear in the reads but are not assembled. The green curve corresponds to *k*-mers shared by both haplotypes, and the red and blue curves show *k*-mers found only in one of the haplotypes.

The combined primary and alternate assemblies achieve an estimated QV of 60.4. The *k*-mer completeness is 73.87% for the primary assembly, 73.02% for the alternate haplotype, and 98.69% for the combined assemblies ( Figure 4).

**Figure 5.**
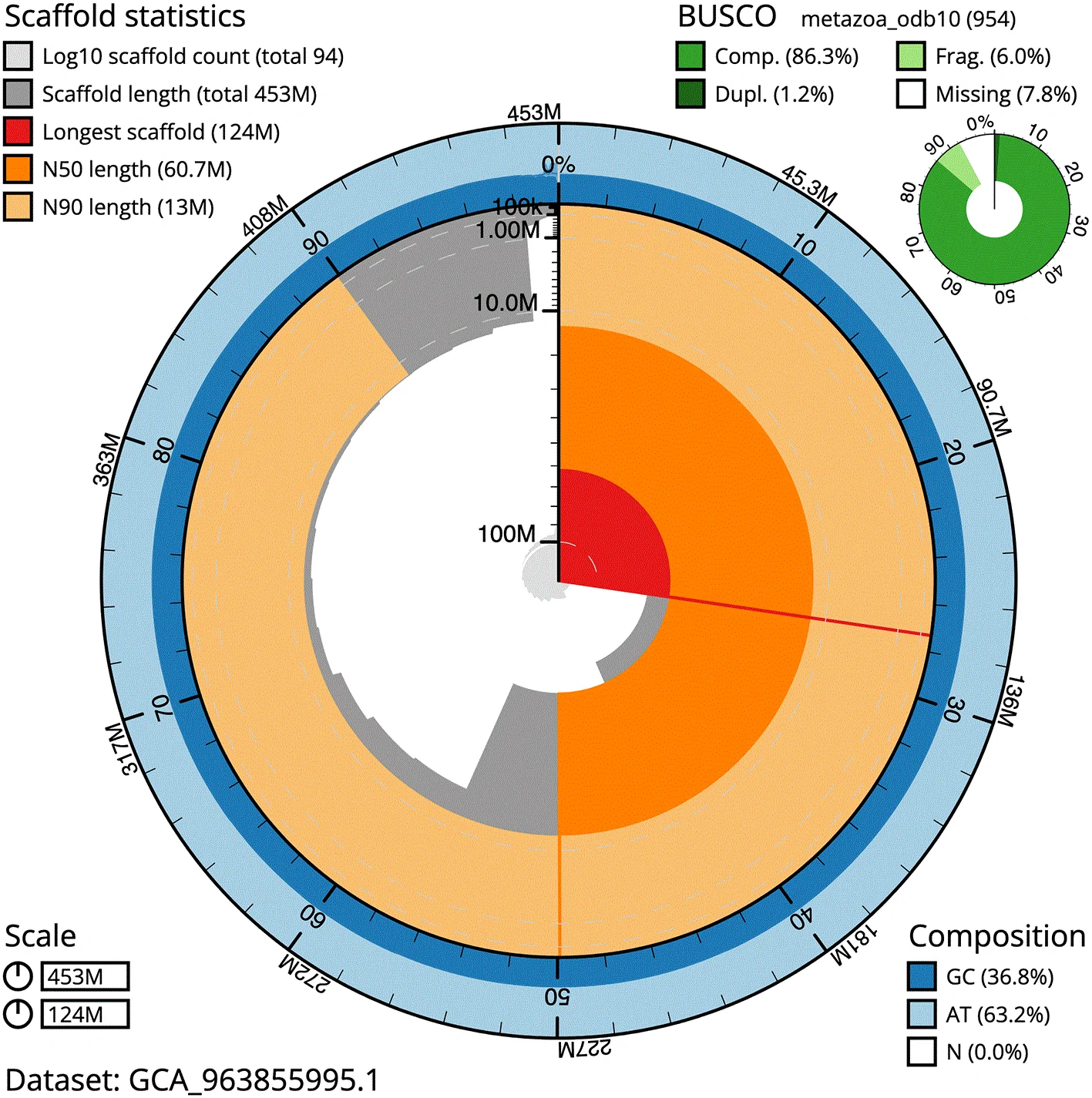
Assembly metrics for jaMurMuri3.1. The BlobToolKit snail plot provides an overview of assembly metrics and BUSCO gene completeness. The circumference represents the length of the whole genome sequence, and the main plot is divided into 1 000 bins around the circumference. The outermost blue tracks display the distribution of GC, AT, and N percentages across the bins. Scaffolds are arranged clockwise from longest to shortest and are depicted in dark grey. The longest scaffold is indicated by the red arc, and the deeper orange and pale orange arcs represent the N50 and N90 lengths. A light grey spiral at the centre shows the cumulative scaffold count on a logarithmic scale. A summary of complete, fragmented, duplicated, and missing BUSCO genes in the set is presented at the top right. An interactive version of this figure can be accessed on the BlobToolKit viewer.

**Figure 6.**
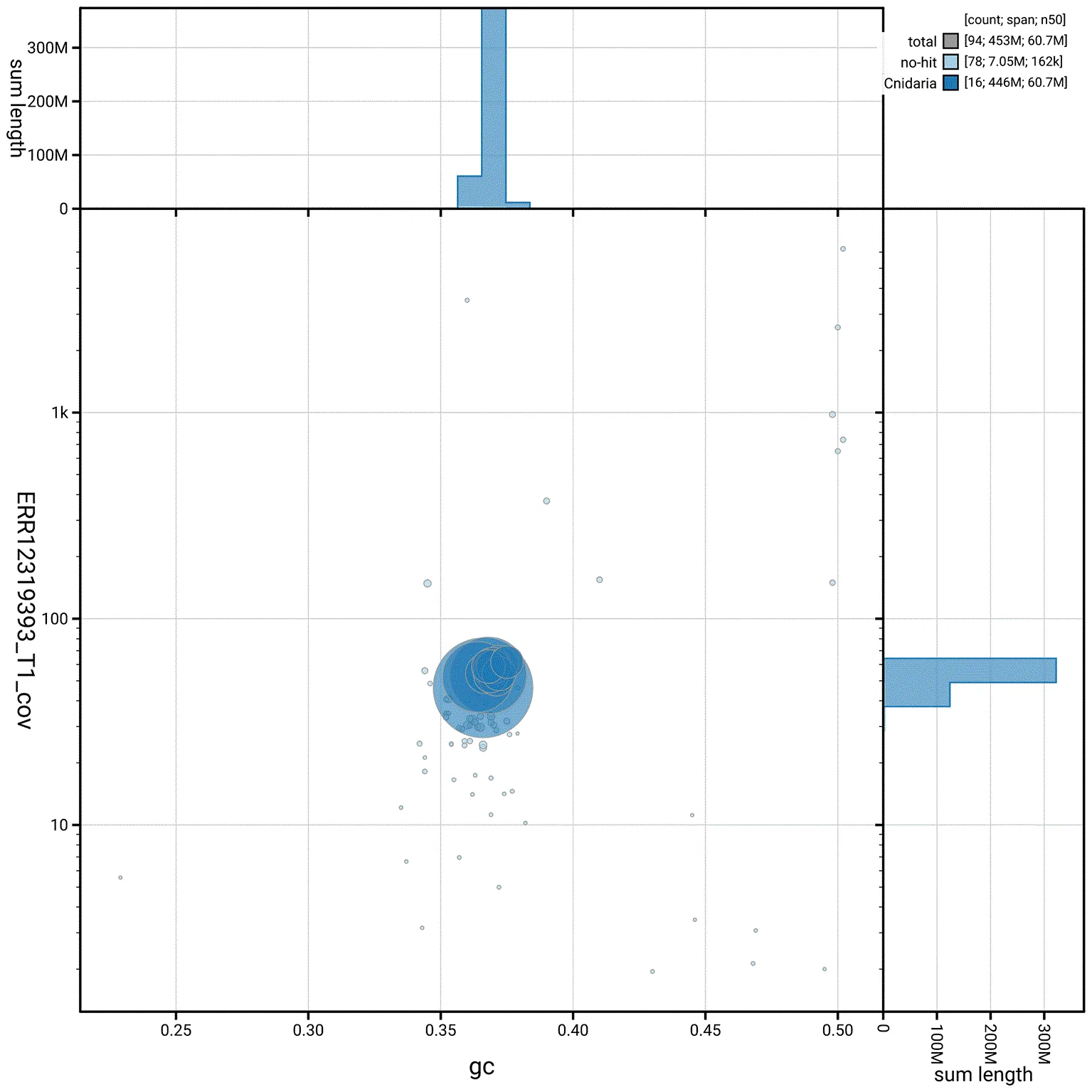
BlobToolKit blob plot for jaMurMuri3.1. The plot shows base coverage (vertical axis) and GC content (horizontal axis). The circles represent scaffolds, with the size proportional to scaffold length and the colour representing phylum membership. The histograms along the axes display the total length of sequences distributed across different levels of coverage and GC content. An interactive version of this figure is available on the BlobToolKit viewer.

BUSCO v.5.4.3 analysis using the metazoa_odb10 reference set ( *n* = 954) identified 86.3% of the expected gene set (single = 85.1%, duplicated = 1.2%). The snail plot in Figure 5 summarises the scaffold length distribution and other assembly statistics for the primary assembly. The blob plot in Figure 6 shows the distribution of scaffolds by GC proportion and coverage.

Table 4 lists the assembly metric benchmarks adapted from Rhie *et al.* (2021) and the Earth BioGenome Project Report on Assembly Standards January 2026. The EBP metric, calculated for primary assembly, is **6.C.Q61**, meeting the recommended 6.C.Q40 reference standard.

**Table 4.** Earth Biogenome Project summary metrics for the *Muricea muricata* assembly.

Measure	Value	Benchmark
EBP summary (primary)	6.C.Q61	6.C.Q40
Contig N50 length	4.02 Mb	≥ 1 Mb
Scaffold N50 length	60.66 Mb	= chromosome N50
Consensus quality (QV)	Primary: 61.5; alternate: 59.7; combined: 60.4	≥ 40
*k*-mer completeness	Primary: 73.87%; alternate: 73.02%; combined: 98.69%	≥ 90%
BUSCO	C:86.3%[S:85.1%,D:1.2%], F:6.0%,M:7.8%,n:954	S > 90%; D < 5%
Percentage of assembly assigned to chromosomes	98.45%	≥ 90%

*Notes:* The EBP summary uses log10(Contig N50); chromosome-level (C) or log10(Scaffold N50); Q (Merqury QV). BUSCO: C = complete; S = single-copy; D = duplicated; F = fragmented; M = missing; n = orthologues

### Genome annotation report

The *Muricea muricata* genome assembly (GCA_963855995.1) was annotated by Ensembl at the European Bioinformatics Institute (EBI). This annotation includes 55 339 transcribed mRNAs from 52 164 protein-coding genes. The average transcript length is 2 970.06 bp, with 4.10 exons per transcript. Further details of this annotation are available from the Ensembl annotation page.

### Metagenome report

**Figure 7.**
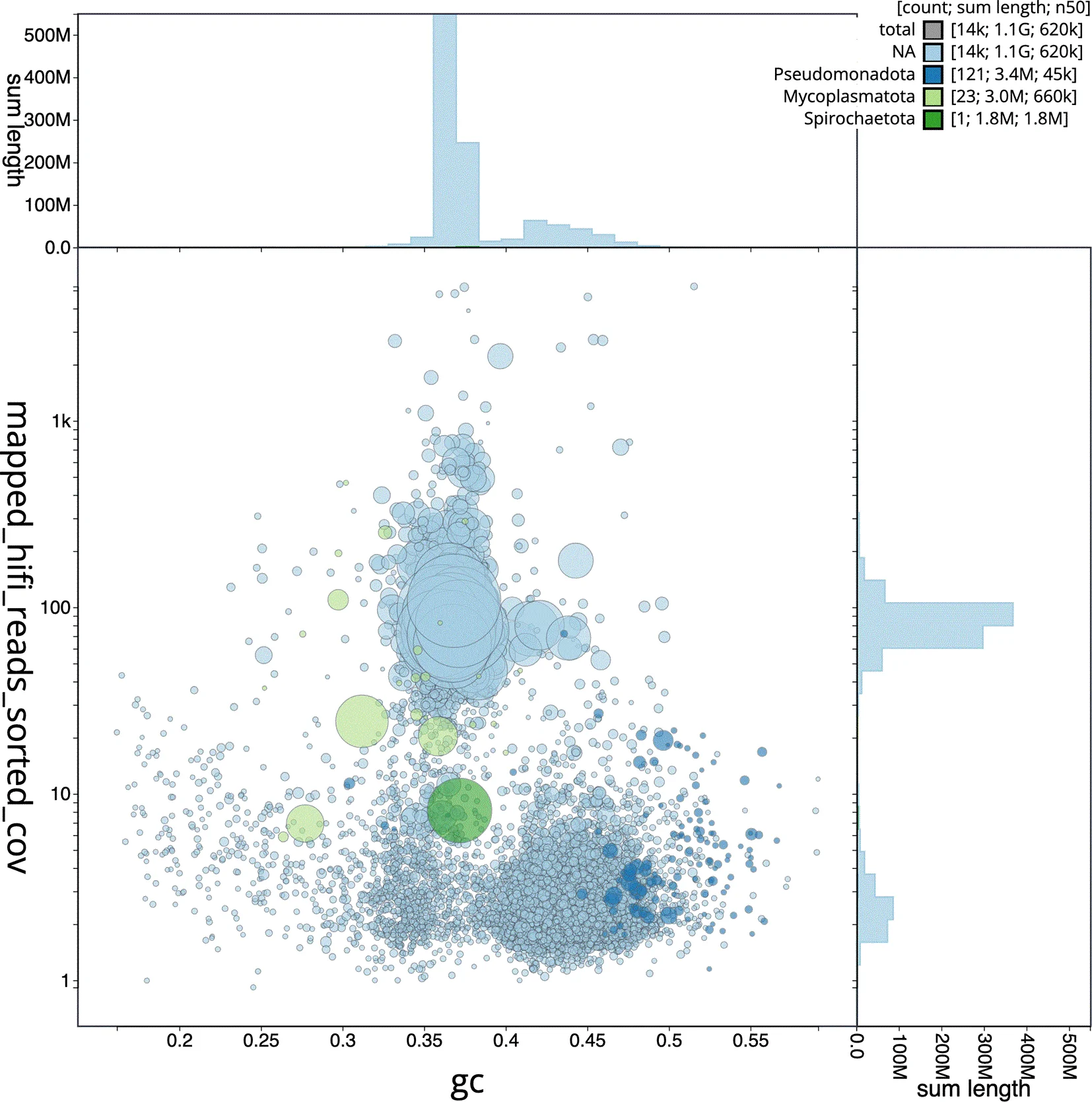
Blob plot for sequences in the *Muricea muricata* metagenome. The plot shows base coverage (vertical axis) and GC content (horizontal axis). Binned contigs are coloured by family. Circles are sized in proportion to sequence length on a square-root scale, ranging from 510 to 6 879 066. Histograms show the distribution of sequence length sum along each axis. An interactive version of this figure may be viewed here.

**Figure 8.**
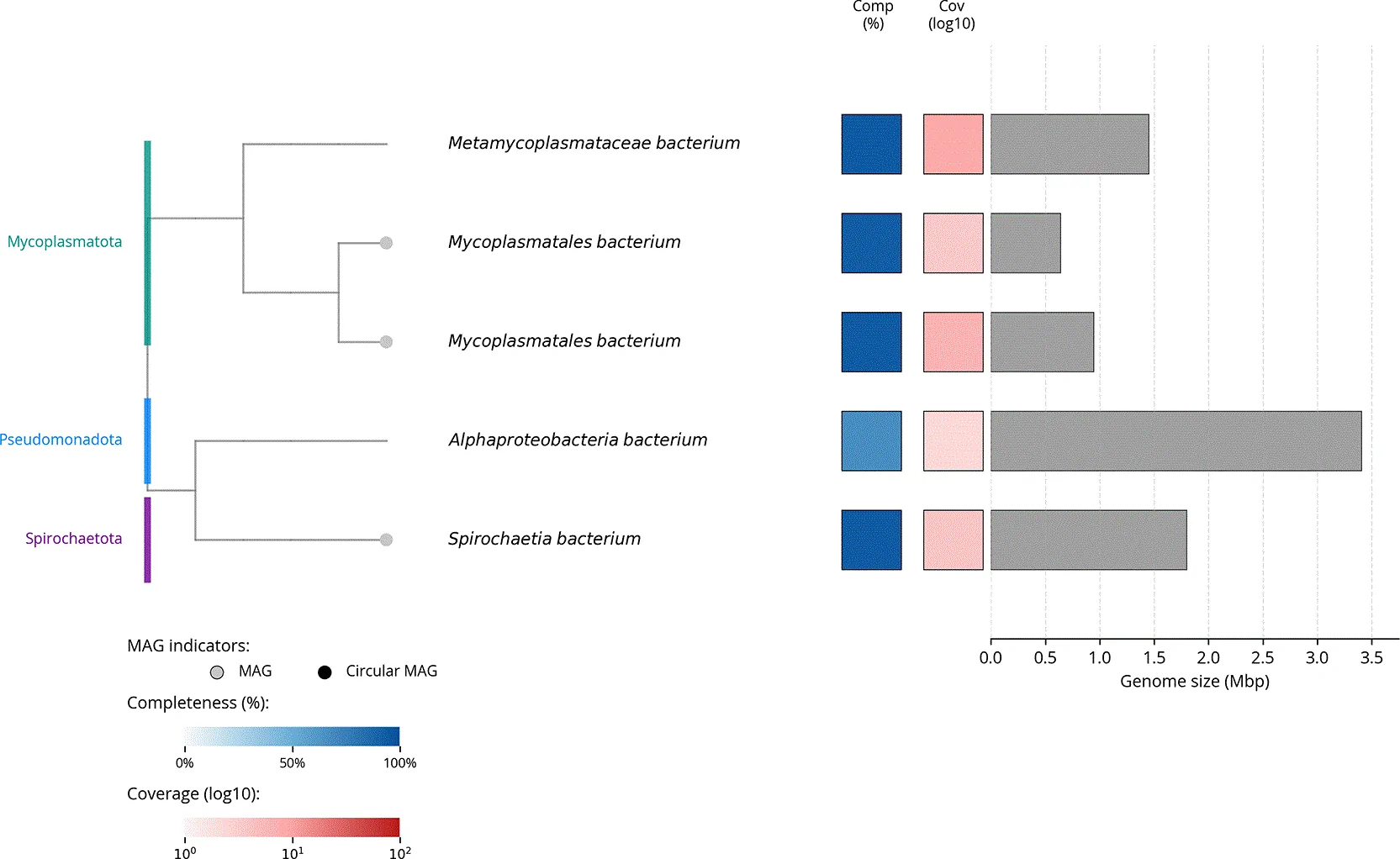
Taxonomic tree based on taxonomic classifications of metagenome bins, constructed using ete3. Colours indicate phylum-level taxonomy. Tracks show genome completeness (blue), sequencing coverage (red, log _10_), and genome size (grey bars, Mbp). High-quality MAGs are marked with grey circles; fully circularised MAGs in black.

We recovered five bins from the metagenome assembly ( Figure 7), of which three met the criteria for MAGs. The recovered bins represented three bacterial phyla, with genome sizes ranging from 0.64 to 3.41 Mbp (mean: 1.65 ± 0.97 Mbp). Mean completeness was 89.1% (± 10.7%) with 0.4% (± 0.4%) contamination. Table 5 and Figure 8 summarise the taxa and quality of the metagenome bins.

**Table 5.** Quality metrics and taxonomic assignments of the binned metagenomes.

NCBI taxon	Taxid	GTDB taxonomy	Quality	Size (bp)	Contigs	Circular	Mean coverage	Completeness (%)	Contamination (%)
Metamycoplasmataceae bacterium	2979199	Bacilli_A	MEDIUM	1452366	11	No	24.16	95.61	0.38
Alphaproteobacteria bacterium	1913988	Alphaproteobacteria	MEDIUM	3409141	121	No	3.57	67.74	1.08
Spirochaetia bacterium	2053615	Spirochaetia	HIGH	1800609	1	No	7.94	94.12	0
Mycoplasmatales bacterium	2023991	Bacilli_A	HIGH	944624	10	No	16.74	94.36	0.75
Mycoplasmatales bacterium	2023991	Bacilli_A	HIGH	641540	2	No	6.51	93.82	0

**Table 6.** Software versions and sources used for *Muricea muricata.*

Software	Version	Source
BLAST	2.14.0	https://ftp.ncbi.nlm.nih.gov/blast/executables/blast+/
BlobToolKit	4.3.3	https://github.com/blobtoolkit/blobtoolkit
BUSCO	5.4.3	https://gitlab.com/ezlab/busco
bwa-mem2	2.2.1	https://github.com/bwa-mem2/bwa-mem2
CheckM	2015-01-16	https://ecogenomics.github.io/CheckM/
DIAMOND	2.1.8	https://github.com/bbuchfink/diamond
dRep	3.4.0	https://github.com/MrOlm/drep
ete3	3.1.3	http://etetoolkit.org
fasta_windows	0.2.4	https://github.com/tolkit/fasta_windows
FastK	1.1	https://github.com/thegenemyers/FASTK
GenomeScope2.0	2.0.1	https://github.com/tbenavi1/genomescope2.0
Gfastats	1.3.6	https://github.com/vgl-hub/gfastats
GTDB-Tk	1.2.1	https://github.com/Ecogenomics/GTDBTk
Hifiasm	0.16.1	https://github.com/chhylp123/hifiasm
HiGlass	1.13.4	https://github.com/higlass/higlass
MAGScoT	1.0.0	https://github.com/ikmb/MAGScoT
matplotlib	3.10.3	https://matplotlib.org/
MerquryFK	1.1.0-c1	https://github.com/thegenemyers/MERQURY.FK
MetaMDBG	Pre-release	https://github.com/GaetanBenoitDev/metaMDBG
Minimap2	2.24-r1122	https://github.com/lh3/minimap2
MitoHiFi	2	https://github.com/marcelauliano/MitoHiFi
MultiQC	1.14; 1.17 and 1.18	https://github.com/MultiQC/MultiQC
Nextflow	23.04.1	https://github.com/nextflow-io/nextflow
PretextSnapshot	0.0.4	https://github.com/sanger-tol/PretextSnapshot
PretextView	1.0.3	https://github.com/sanger-tol/PretextView
Prokka	1.14.5	https://github.com/tseemann/prokka
purge_dups	1.2.3	https://github.com/dfguan/purge_dups
samtools	1.16.1; 1.17	https://github.com/samtools/samtools
sanger-tol/ascc	0.1.0	https://github.com/sanger-tol/ascc
sanger-tol/blobtoolkit	0.3.0	https://github.com/sanger-tol/blobtoolkit
sanger-tol/curationpretext	1.4.2	https://github.com/sanger-tol/curationpretext
Seqtk	1.4-r122	https://github.com/lh3/seqtk
Singularity	3.9.0	https://github.com/sylabs/singularity
TreeVal	1.0.0	https://github.com/sanger-tol/treeval
YaHS	1.1a.2	https://github.com/c-zhou/yahs

## Author information

Contributors are listed at the following links:

• Members of the Wellcome Sanger Institute Tree of Life Management, Samples and Laboratory team
• Members of Wellcome Sanger Institute Scientific Operations – Sequencing Operations
• Members of the Wellcome Sanger Institute Tree of Life Core Informatics team
• Members of the EBI Aquatic Symbiosis Genomics Data Portal Team
• The Aquatic Symbiosis Genomics Project leadership

## Wellcome Sanger Institute – Legal and Governance

The materials that have contributed to this genome note have been supplied by a Tree of Life collaborator. The Wellcome Sanger Institute employs a process whereby due diligence is carried out proportionate to the nature of the materials themselves, and the circumstances under which they have been/are to be collected and provided for use. The purpose of this is to address and mitigate any potential legal and/or ethical implications of receipt and use of the materials as part of the research project, and to ensure that in doing so we align with best practice wherever possible.

The overarching areas of consideration are:

• Ethical review of provenance and sourcing of the material
• Legality of collection, transfer and use (national and international)

Each transfer of samples is undertaken according to a Research Collaboration Agreement or Material Transfer Agreement entered into by the Tree of Life collaborator, Genome Research Limited (operating as the Wellcome Sanger Institute) and in some circumstances other Tree of Life collaborators.

## Data Availability

European Nucleotide Archive: Muricea muricata (spiny sea fan). Accession number PRJEB70484; BioProject URL: https://identifiers.org/ena.embl/PRJEB70484. The genome sequence is released openly for reuse. The *Muricea muricata* genome sequencing initiative is part of the Aquatic Symbiosis Genomics Project (PRJEB43743) and the Sanger Institute Tree of Life Programme (PRJEB43745). All raw sequence data and the assembly have been deposited in INSDC databases. Raw data and assembly accession identifiers are reported in Tables 1 and 2. Production code used in genome assembly at the WSI Tree of Life is available at https://github.com/sanger-tol . Table 6 lists software versions used in this study.
