# Supplementary material for: The chromosomal genome sequence of the spiny sea fan, Muricea muricata (Pallas, 1766) (Malacalcyonacea: Plexauridae) and its associated microbial metagenome sequences
Source: Wellcome Open Res. 2026 Aug 8;11:512. [Version 1] doi: 10.12688/wellcomeopenres.27232.1 (PMC13560551; doi:10.12688/wellcomeopenres.27232.1)
Supplement: Peer review history

# Reviewer report: Reviewer response for version 1

**DOI:** 10.21956/wellcomeopenres.29972.r164117

**Published:** 2026-08-31

**Authors:** Alexander Klimovich

**Affiliations:** 1 Christian-Albrechts University of Kiel, Kiel, Germany

This Data Note present a chromosome-level genome assembly for the octocoral *Muricea muricata* along with basic assembly statistics and gene annotation. Additionally, it provides an assembly of associated microbial sequences. The dataset is a valuable addition to octocoral genomic resources and will greatly support future work in symbiosis research, comparative genomics, and coral conservation and restoration.

 The rationale for generating the resource is well articulated, and the methods section is exemplary in its level of detail - it sets a truly high standard for transparent reporting of analytical steps in reference genome publications. The methodology as described is sound and appropriate for the aims.

**Suggestions for improvement:**

 I have three minor points that I believe would strengthen the Data Note; none require substantial additional analysis.

1. The authors could briefly cover the current state of genomic resources in related taxa. To my knowledge, no high-quality genome assemblies currently exist for other *Muricea* species, or indeed for the family Plexauridae more broadly. Making this explicit would help the authors better articulate the novelty and value of this dataset.
2. It is somewhat surprising that no *Breviolum* genome was recovered alongside the coral host and microbial genomes, given that this representative of Symbiodiniaceae is consistently associated with the spiny sea fan. If the apo symbiotic tissue from a symbiont-free bleached colony was used for gDNA extraction, this is worth being mentioned in the Methods. If the dinoflagellate sequences (nuclear, mitochondrial and plastid genomes) were computationally filtered out during assembly - this is also worth mentioning. However, if neither applies and *Breviolum* genome sequence is in fact present in the raw data, including it as an additional resource would add considerable value, given its role as a core holobiont member.
3. As the genome and transcriptome data appear to derive from different samples, it would strengthen the microbial component of the paper to state whether the RNA-seq data contain transcripts mapping to the three recovered bacterial MAGs. This would support a stable, functional association rather than incidental presence. Also, if data on relative microbial density (e.g., bacterial genome copies per single copy host nuclear locus) are available, including this would help readers assess whether these MAGs represent stable and abundant holobiont members or represent rather an accidental contamination. It appears that the samples were not rinsed prior to gDNA extraction, so contamination with bacteria from the environment or the mucus is likely.

Minor comment

 Please check the spelling of the dinoflagellate symbiont genus in the Background section. It should read *Breviolum* (not *Brevolium*).

Are sufficient details of methods and materials provided to allow replication by others?

Yes

Is the rationale for creating the dataset(s) clearly described?

Yes

Are the datasets clearly presented in a useable and accessible format?

Yes

Are the protocols appropriate and is the work technically sound?

Yes

Reviewer Expertise:

Evolutionary developmental biology, genomics, host-microbiome interactions

I confirm that I have read this submission and believe that I have an appropriate level of expertise to confirm that it is of an acceptable scientific standard.

# Reviewer report: Reviewer response for version 1

**DOI:** 10.21956/wellcomeopenres.29972.r164120

**Published:** 2026-08-28

**Authors:** Yuki Yoshioka

**Affiliations:** 1 Okinawa Institute of Science and Technology Graduate University, Onna, Japan

The authors provide a high-quality genome assembly of the spiny sea fan, *Muricea muricata*, along with its associated microbial metagenomic sequences. The authors used well-established pipelines to generate these datasets, and the resulting quality meets the reported metrics. In general, I have only a few comments and found several errors in the text.

 (1) “genus *Brevolium*” is incorrect. It should be *Breviolum*.

 (2) How do the authors distinguish between “ *M. muricata*” and “ *Muricea muricata*” in this manuscript? The manuscript inconsistently switches between the full binomial "Muricea muricata" and the abbreviated "M. muricata" within the Background section (e.g., full form in paragraph 1, abbreviated in paragraph 2, full form again in paragraph 3, abbreviated in paragraph 4, and full form again in paragraph 5). Per standard convention, the abbreviated form should be used consistently after the first mention. Please revise for consistency throughout the manuscript.

 (3) GenomeScope estimated the genome size to be 782 Mb, whereas the final assembly is only 453 Mb. It would be helpful to discuss this discrepancy, as also suggested by another reviewer.

 (4) The authors cited GTDB-Tk v2, but the version reported in Table 6 is 1.2.1. It would be better to check the version carefully and ensure that the information is consistent throughout the manuscript.

Are sufficient details of methods and materials provided to allow replication by others?

Yes

Is the rationale for creating the dataset(s) clearly described?

Yes

Are the datasets clearly presented in a useable and accessible format?

Yes

Are the protocols appropriate and is the work technically sound?

Yes

Reviewer Expertise:

Cnidarian genomics

I confirm that I have read this submission and believe that I have an appropriate level of expertise to confirm that it is of an acceptable scientific standard.

# Reviewer report: Reviewer response for version 1

**DOI:** 10.21956/wellcomeopenres.29972.r164119

**Published:** 2026-08-26

**Authors:** Qingxiang Guo

**Affiliations:** 1 Northwestern University Feinberg School of Medicine, Chicago, Illinois, USA

This Data Note presents a chromosome-level genome assembly for the spiny sea fan Muricea muricata, along with genome annotation and microbial metagenome data from the same study. The resource should be useful for future work on octocoral genomics and related comparative studies. The manuscript is mostly straightforward, although several parts of the data description and analysis (assembly quality, sequencing depth, metagenome workflow) need to be clarified before the quality and reproducibility of the resource can be fully judged.

 “As with other plexaurids, M. muricata has garnered attention in recent years due to its chemical ecology and adaptive mechanisms. Researchers have identified bioactive compounds within this species that may have pharmaceutical applications, including anti-inflammatory and antimicrobial properties (Camacho et al., 2014).”

 - Camacho et al. (2014) appears to report results for Muricea sp., rather than M. muricata specifically. The current wording therefore seems to extend genus-level evidence to this particular species. Unless the authors can provide direct evidence for M. muricata, I suggest revising this sentence to state that bioactive compounds have been reported in the genus Muricea, rather than saying that they have been identified in “this species.”

 “The nuclear genome assembly of Muricea muricata presented here could …”

 - I think the authors should briefly place this new genome in the context of existing genomic resources. The Background explains why M. muricata is ecologically interesting, but gives little information on what genomes are already available for Muricea, Plexauridae, or closely related octocorals. A short paragraph on the current genomic landscape would make it clearer what gap this assembly fills and how it may be useful for future comparative studies.

 “The metagenome assembly was generated using MetaMDBG (Benoit et al., 2024). The resulting bin sets of each binning algorithm were optimised and refined using MAGScoT…”

 - The source of the reads used for the metagenome assembly is not clear. Were all PacBio HiFi reads from jaMurMuri3 used directly, or was MetaMDBG run on a host-depleted set of reads, for example reads removed during the ASCC decontamination step? The authors should describe how the microbial reads were separated from the M. muricata reads before metagenome assembly. This information is important for understanding and reproducing the metagenome analysis.

 “GenomeScope2.0 analysis estimated the haploid genome size at 782.95 Mb, with a heterozygosity of 0.23% and repeat content of 76.28%.”

 - There is a large difference between the GenomeScope2 haploid genome-size estimate of 782.95 Mb and the final primary assembly size of 453.40 Mb. I think this difference needs to be explained, especially since the primary assembly has a k-mer completeness of 73.87% and a BUSCO completeness of 86.3%. The authors should discuss whether the GenomeScope estimate may be inflated by cobiont or microbial sequences, whether highly repetitive regions may have been collapsed or lost during assembly, or whether other filtering or curation steps account for the difference. In this context, the statement that 98.45% of the assembly is assigned to chromosomes should also be clearly distinguished from completeness of the expected genome.

 “PacBio sequencing of the Muricea muricata specimen generated 144.63 Gb…” and “GenomeScope2.0 analysis estimated the haploid genome size at 782.95 Mb…” and “Based on the estimated genome size, the sequencing data provided approximately 118 coverage.”

 - The reported sequencing depth does not seem to match the numbers given in the manuscript. A total of 144.63 Gb of HiFi sequence divided by the estimated genome size of 782.95 Mb gives approximately 185× coverage, rather than 118×. The authors should check this calculation. If the 118× value was calculated from a filtered subset of host reads rather than the full 144.63 Gb, please report the amount of sequence used for that calculation so that the coverage estimate is clear.

 “One of these samples was used for genome sequencing (specimen ID NSU0025901, ToLID jaMurMuri3). A second sample was used for Hi-C sequencing (specimen ID NSU0025902, ToLID jaMurMuri4). The jaMurMuri4 specimen was also used for RNA sequencing.”

 - The PacBio genome was generated from jaMurMuri3, whereas the Hi-C data used for scaffolding and the RNA-seq data came from a second specimen, jaMurMuri4. Table 1 also identifies them as different source individuals. The authors should provide a little more information on the relationship between these two specimens. Since Hi-C data from one individual were used to scaffold the genome assembly of another, it would also be useful for the authors to mention whether any structural differences or unusual Hi-C contacts between the two specimens were noticed during curation.

Are sufficient details of methods and materials provided to allow replication by others?

Partly

Is the rationale for creating the dataset(s) clearly described?

Partly

Are the datasets clearly presented in a useable and accessible format?

Yes

Are the protocols appropriate and is the work technically sound?

Partly

Reviewer Expertise:

Genomics, bioinformatics, long-read sequencing, and genome analysis.

I confirm that I have read this submission and believe that I have an appropriate level of expertise to confirm that it is of an acceptable scientific standard, however I have significant reservations, as outlined above.

# Reviewer report: Reviewer response for version 1

**DOI:** 10.21956/wellcomeopenres.29972.r164122

**Published:** 2026-08-24

**Authors:** Sérgio N. Stampar

**Affiliations:** 1 São Paulo State University (UNESP), Bauru, Brazil

The manuscript presents a high-quality, chromosome-level reference genome assembly for the Caribbean octocoral Muricea muricata, alongside its organellar genome and five associated microbial metagenome-assembled bins.

 There are only two important points to discuss. 1) The BUSCO assessment against "metazoa_odb10" reported 86.3% complete orthologs, with 7.8% missing and 6.0% fragmented. It's typical for non-model marine invertebrates due to evolutionary divergence from standard metazoan lineages, I think that a specialized anthozoan markers could provide deeper verification.  

 2) The identified 52,164 protein-coding genes represents a large gene set. This could reflect real biological gene family expansions (e.g., involved in bioactive secondary metabolite synthesis or thermal stress response) or potential over-prediction by automated annotation pipelines on complex/repetitive genomic regions. It's relevant to discuss. 

 In general, the manuscript delivers a benchmark-quality genomic resource that meets international Earth BioGenome Project standards. It fills a critical gap in octocoral genomics, establishing a foundation for research into coral thermal tolerance, bleaching resilience, chemical ecology, and host-microbiome dynamics in Caribbean reef ecosystems.

Are sufficient details of methods and materials provided to allow replication by others?

Yes

Is the rationale for creating the dataset(s) clearly described?

Yes

Are the datasets clearly presented in a useable and accessible format?

Yes

Are the protocols appropriate and is the work technically sound?

Yes

Reviewer Expertise:

Marine Evolution; Cnidaria

I confirm that I have read this submission and believe that I have an appropriate level of expertise to confirm that it is of an acceptable scientific standard.
